# Treatment utilization and barriers to treatment: Results of a survey of dependent methamphetamine users

**DOI:** 10.1186/1747-597X-6-3

**Published:** 2011-02-14

**Authors:** Pauline Kenny, Angela Harney, Nicole K Lee, Amy Pennay

**Affiliations:** 1 Turning Point Alcohol and Drug Centre, 54-62 Gertrude Street, Fitzroy, Victoria, 3065, Australia; 2 School of Psychology, Psychiatry and Psychological Medicine, Monash University, Clayton, Victoria, Australia; 3 Eastern Health Clinical School, Monash University, Clayton, Victoria, Australia; 4 National Centre for Education and Training on Addictions, Flinders University, Adelaide, Australia

## Abstract

**Background:**

Australia has one of the highest rates of methamphetamine use in the world; however, treatment access for methamphetamine is comparatively low. This descriptive study aimed to identify patterns of treatment utilization and perceived barriers to accessing treatment among dependent methamphetamine users in the hope that such information will enable services to more appropriately respond to this group.

**Methods:**

One hundred and twenty-six methamphetamine users who had a current or past history of methamphetamine dependence were interviewed about their experiences of, and perceived barriers to, treatment.

**Results:**

Treatment utilization among methamphetamine users was reportedly low. One of the main reasons cited for not accessing treatment was that methamphetamine users did not perceive their drug use to be a problem (despite apparent levels of dependence). Self-detoxification with the use of other licit and illicit drugs was high among this group. Participants identified a lack of confidence in the ability of treatment services to address methamphetamine dependence and the 'opiate-centric' nature of treatment services as significant blocks to treatment entry. Suggestions for improvement by participants included operating specialist services for methamphetamine users, placing an emphasis on responsiveness and routinely involving case management services for this group.

**Discussion and Conclusions:**

To improve service delivery, treatment services should reorient their services to better address the needs of methamphetamine users by making small changes such as specific opening times for methamphetamine users or using a dedicated space for methamphetamine treatment. Alternative options such as online treatments and specialist methamphetamine clinics should be considered for methamphetamine users.

## Introduction

The use of methamphetamine is of significant concern to the community. Australia has one of the highest rates of methamphetamine use in the world with over 6% of the population reporting having tried the drug and over 2% currently using [1]. Australia also has one of the highest rates of methamphetamine injecting in the world, bringing with it additional harms and higher risk of dependence [2].

There are estimated to be over 73,000 dependent methamphetamine users in Australia [3], making up approximately 11% of regular users. Yet treatment utilization among methamphetamine users is relatively low [4]. For example in Australia, methamphetamine accounts for only 11.2% of total treatment presentations, behind alcohol (44.5%) and cannabis (21.6%). Further, methamphetamine has around the same number of treatment presentations as heroin (10.5%) despite having more than twice the number of dependent users [3,4].

The reasons for low treatment seeking among methamphetamine users is not clear, but authors have suggested a number of potential reasons including the poor orientation of services to this group, lack of information about treatment options and little confidence in the effectiveness of these programs [5].

Lee and colleagues [6] have found that methamphetamine users can wait an average of five years from first experiencing problems with their methamphetamine use and seeking treatment. Other research has found that when methamphetamine users do seek treatment, there is a substantial likelihood of treatment dropout or relapse [7].

While there has been some recent research into barriers to treatment for illicit drug users more broadly, there has been very little research on barriers specific to methamphetamine treatment, particularly in Australia. Barriers to effective service delivery for illicit drugs users more generally include social stigma, perceptions about treatment effectiveness, accessibility and long waiting periods [8-12]. Barriers specific to methamphetamine use, which were reported over a decade ago in the UK, include methamphetamine users not identifying themselves as 'hard' or dependent drug users and being unwilling to mix with heroin users [13]. Indeed, over ten years ago, 65% of methamphetamine users in the UK indicated that they perceived treatment services to be inappropriate for them and not meeting their particular needs [14].

Pennay and Lee [15] recently identified barriers to methamphetamine withdrawal treatment from the service provider perspective, noting that service providers saw barriers to methamphetamine treatment as extensive and wide-ranging. Some of these barriers included: particular personality characteristics of methamphetamine users, complexities associated with mental health comorbidity, waiting periods resulting in loss to treatment, the binge nature of methamphetamine use complicating withdrawal, lacking appropriate pharmacotherapy options and negative attitudes of staff towards this group. However, this study was from a service perspective view and focused only on withdrawal treatment.

Through interviews with 126 dependent methamphetamine users, the current study aimed to identify patterns of treatment utilization and perceived barriers to accessing treatment. By developing a comprehensive understanding about the problems associated with accessing treatment from the perspective of methamphetamine users, treatment services might be able to better orient their services to overcome such barriers and in turn, improve engagement and retention.

## Methods

### Participants

One hundred and twenty-six adult illicit methamphetamine users who met DSM-IV [16] criteria for past or present methamphetamine dependence were interviewed about their treatment utilization, experiences of treatment and perceived barriers to treatment. Males comprised 71% (n = 89) of the sample, the average age was 32 years (SD = 8.6; range: 19-59 years) and 84% (n = 106) were Australian born. Fifty-seven percent (n = 72) had either no qualifications or secondary school only and 66% (n = 83) were unemployed at the time of the survey.

Eighty-six percent (n = 102 of 119) of participants identified their time of most frequent methamphetamine use as 'in the past', ranging from one month to 28 years prior to the time of interview (mean = 59.8 months; SD = 65.0). Eighty percent (n = 101) of participants had used methamphetamine in the six months prior to the interview, while 68% (n = 86) had used in the month prior. Forty-two percent (n = 53) reported using methamphetamine on at least four days in that month.

Sixty percent of the sample (n = 76) reported the age of first use of methamphetamine between 15 and 18 years, with 43% snorting (n = 54) and 36% (n = 45) injecting as their first route of administration. Eighty percent (n = 101) had injected methamphetamine and the mean Severity of Dependence score was 8.2 (SD = 2.5; range 4-14 years), indicating high levels of dependence [17].

Methamphetamine was the most commonly cited drug of choice (n = 43; 34%), but high levels of polydrug use were evident in the sample. The substance used *most often *by participants in the six months prior to interview were methamphetamine (n = 39; 31%), cannabis (n = 26; 21%), heroin (n = 18; 14%) and alcohol (n = 15; 12%).

Previous experience of specialist alcohol and other drug (AOD) treatment not related to methamphetamine use was high (n = 74; 59%) and 40% (n = 50) of participants reported current engagement with AOD treatment. The most common current treatment was opioid pharmacotherapy programs (n = 33; 26%), followed by counseling (n = 18; 14%).

### Measures

A structured questionnaire delivered via notebook computer using specifically designed software [18] asked about a range of demographic variables and used a combination of scaled response, multiple choice and open-ended questions about users' experiences and attitudes towards treatment.

The Severity of Dependence Scale (SDS) [17] was used as a preliminary screening tool to indicate participant eligibility for past or present methamphetamine dependence. The SDS is a brief instrument that can be administered over the telephone. A cut-off of four as suggested by Topp and Mattick [19] was used as an indication of past or present dependence. During the interview, the Structured Clinical Interview for DSM-IV-TR (SCID-I) [20], a semi-structured interview for making the major DSM-IV Axis I diagnoses, was administered as a more rigorous measure of methamphetamine dependence during the time of the participants' most frequent use of the drug. While completing the survey tool, participants were asked to reflect on their most frequent period of methamphetamine use.

### Procedure

Participants in metropolitan Melbourne were recruited over 12 months from community health and specialist AOD programs, public health services such as needle and syringe programs, and the general public via advertising in street press and online forums. Participants were first screened over the telephone for eligibility using the SDS. Eligible participants then met with a researcher at a convenient and private location to complete the questionnaire. The majority of the questionnaire could be self administered, and responses were recorded directly onto a laptop computer. Self administration was dependent on the participants' level of literacy, computer skill and/or confidence. When necessary or requested, the researcher read out sections and entered responses with participant's permission. The SCID-I dependence measures were researcher-administered only. The structured computer-delivered interviews took around one hour, but on occasion took as long as two hours. Participants were reimbursed $30 for their time. Ethics approval was granted for the study from the Human Research Ethics Committee of the Department of Human Services (now Department of Health), Victoria, Australia. All procedures followed were in accord with the standards of this committee.

Quantitative data was analyzed using SPSS version 14.0 and primarily involved descriptive and bivariate analysis. Qualitative responses were extracted from SPSS, and coded according to common themes. For the majority of data reported, no more than 1-2% was missing and has been treated as missing data in descriptive analysis. Reduced response rates are evident for some variables, due to questionnaire sections directed only at sub-samples of participants and resulting from participants who felt unable to respond ('don't know' or 'refuse to answer' responses). The frequency of the latter may have been increased due to self-administration of the questionnaire. When significant, these reduced response samples are indicated in the text.

## Results

### Treatment seeking among methamphetamine users

Based on a five point scale, 49% (n = 62) of participants reported being well to very well informed about the available options for methamphetamine treatment. A further 37% (n = 46) indicated being moderately informed.

Despite being well informed and although 65% (n = 80) of the sample had ever perceived a need for treatment to help with withdrawal, only 34% (n = 43) had actually sought treatment for their methamphetamine use. Counseling was the most frequently accessed treatment by the sample (n = 28; 22%), followed by inpatient withdrawal (n = 19; 15%) and Narcotics Anonymous (n = 14; 11%).

Table 1 shows participants' awareness of different AOD treatment options, and past utilization of treatment types specific to managing methamphetamine withdrawal.

**Table 1:** Awareness of AOD specific treatment options and treatment utilization for methamphetamine withdrawal management

Treatment option	Treatment awareness n (%) *(n = 126)*	Treatment utilization n (%) *(n = 125)*
Drug counseling	121 (96%)	28 (22%)

Narcotics Anonymous	113 (90%)	14 (11%)

Residential/inpatient withdrawal	98 (78%)	19 (15%)

Outpatient withdrawal	73 (58%)	5 (4%)

General practitioner (GP)^a^	n/a	9 (7%)

Therapeutic community^b^	n/a	8 (6%)

^a^GP (not AOD specific)

^b^Awareness of therapeutic communities was not asked

Participants reported self-detoxifying from methamphetamine more often than seeking formal treatment. Of 122 respondents to a series of closed category questions, 94% (n = 115) reported that most withdrawal attempts were without professional support and 79% (n = 96) typically managed withdrawal from methamphetamine by using other illicit drugs, alcohol or prescription medications to deal with symptoms. The sample had previously undergone withdrawal using other drugs an average of 10.4 times (SD = 19.4), and four times 'cold turkey'.

### Reasons for treatment seeking

The 43 participants who had sought formal treatment were asked their reasons (using a multiple response list of options). Over two-thirds (n = 28) identified their primary reasons for accessing treatment as being sick of the lifestyle, pressure from friends and family, and seeking abstinence (Table 2).

**Table 2:** Reasons for seeking methamphetamine treatment

Reasons for seeking treatment	n (%) *(n = 43)*
Sick of the lifestyle	28 (65%)

Pressure from family and friends	28 (65%)

Seeking abstinence	21 (49%)

Financial reasons	17 (40%)

Wanted 'time out'	13 (30%)

Legal reasons	13 (30%)

Health problems	10 (23%)

Wanted to reduce use	9 (21%)

Responses to a series of open ended questions found that among those who had sought methamphetamine treatment, one third (n = 14) had criticisms of treatment, including difficulty with access, dissatisfaction with the medication regime and poor treatment from staff. A further nine participants referred to issues such as the negative impact of treatment on their work, family relationships and finances.

### Barriers to methamphetamine treatment seeking

Thirty five percent of the sample (n = 43) reported they had never felt the need to access formal treatment. The primary reasons (drawn from a list of multiple responses) included that they did not consider their drug use to be serious enough (n = 16), they did not consider formal treatment necessary (n = 13) or they did not consider their drug use to be a problem (n = 10).

Of those who had ever felt a need for methamphetamine withdrawal treatment (n = 80), 83% (n = 67) reported ever having a time when they felt a need for help but did not attempt to seek it. This group was asked to identify treatment barriers from an extensive multiple response list (including the option to specify 'other'). The main reasons preventing them from accessing treatment included: the desire to attempt withdrawal on their own (n = 39; 57%), not knowing how to go about accessing treatment (n = 28; 41%) and wanting to keep using methamphetamine (n = 26; 38%) (Table 3).

**Table 3:** Barriers to methamphetamine treatment seeking

Barriers	n (%) *(n = 68)*
Wanted to attempt to withdraw on own	35 (52%)

Didn't know how to access treatment	28 (41%)

Wanted to keep using	26 (38%)

Unaware of the available treatment options	26 (38%)

Embarrassed or felt stigma attached to treatment	22 (32%)

Lack of support	21 (31%)

Cost/financial difficulties	14 (21%)

Work commitments	13 (19%)

Too hard to get into treatment	13 (19%)

Waiting lists too long/didn't want to wait	12 (18%)

Little confidence in effectiveness	8 (12%)

Treatment goals not compatible with personal goals	8 (12%)

### Suggested improvements for methamphetamine treatment

A small group of participants with no previous methamphetamine treatment experience and who reported they would not contemplate going to an AOD treatment service (n = 20) were asked what would attract them into methamphetamine treatment. Response to this multiple choice question (including an open ended 'other' option) found nearly half (n = 9) called for effective counseling; seven participants wanted a pharmacotherapy to assist in the management of withdrawal, and six reported that a specialist center for methamphetamine users would be the only thing that would attract them into treatment.

The need for specialist methamphetamine centers was highlighted in participants' open ended responses to their attitudes to, and perception of, currently available formal treatment options. Of 121 respondents, 22 people referred directly to a lack of stimulant focused or appropriate treatment and/or lack of treatment option information available, as demonstrated in the following verbatim examples:

*"I think that there are a lot of things lacking in terms of what is available for speed *[methamphetamine]*. It is all geared toward heroin and it just isn't good enough. It is a very different detox and staff aren't trained to know what to expect"*

*"Generally, I think they *[treatment services] *are very poor - I guess that's understandable because they are mainly targeted at heroin addicts. There's a change in drug taking habits and the options aren't there for other drugs"*

### Features of a responsive alcohol and drug treatment service

The key features of what constitutes a responsive AOD service were described by the sample and responses were coded into predominant themes (Table 4).

**Table 4:** Key features of a good AOD service

Key Features	n % *(n = 70)*
Supportive staff	39 (56%)

Location (i.e., accessible by public transport, not too far to travel)	15 (21%)

Suitable environment (i.e., calm, clean, quiet)	12 (17%)

Accessible opening hours (i.e., including outside of business hours: evenings and weekends)	12 (17%)

Staff personally experienced with drug use	11 (16%)

Case management	9 (13%)

Immediate/quick appointment (i.e., no waiting lists)	9 (13%)

Accurate information made available	5 (7%)

Individually tailored treatment, with multiple treatment options and client input	5 (7%)

Of those participants who responded to the question (n = 70), over 50% (n = 39) referred to the importance of appropriate staff and their characteristics as a feature of a good AOD service. Respondents highlighted the need for staff that are friendly, supportive, non-judgmental, compassionate, well trained and knowledgeable. Staff that understand methamphetamine use and have experience with methamphetamine users was important, with 16% (n = 11) of respondents recognizing the benefits of AOD workers who had personal experience with their own drug use, or at least general life experience and understanding of drug use and drug users.

While 21% (n = 15) of respondents highlighted location as an important feature, these were divided into those who believed an isolated location was beneficial and those who felt a metropolitan location was important.

Accessibility was cited as important factors by a number of respondents (17%, n = 12), with one participant saying:

"It needs to be free, accessible, appointment quickly, opening hours - shouldn't have to wait too long to see someone. When you're going through these periods, you don't need another excuse not to go... At your first point of contact even if you have a negative experience at the counter, first person you talk to on the phone, the first person you talk to is so important. The smallest little f**k up on their part will send you running straight back to the people who got you there in the first place"

Some respondents also identified the need for case management as part of drug treatment (13%; n = 9), noting that their primary needs took precedence (i.e., access to safe and stable accommodation, regular income and life skills), and they would not be prepared to engage in specific treatment for their methamphetamine dependence until such factors were in control. For example:

"Helping people to get some goals and having something to look forward to in life... Ask them what they want to achieve and give them things to work forward to. When they see some progress, then it's like savings - you get motivated to work harder to get there"

## Discussion and Conclusions

Research over a decade ago in the UK highlighted both the low treatment attendance and perceived ineffectiveness of methamphetamine treatment [13,14]. Today, methamphetamine treatment access in Australia is relatively low [4], and findings from this study indicate that methamphetamine users perceive a number of barriers to methamphetamine treatment. The current study provides a snapshot of views about drug treatment and barriers to treatment from the perspective of methamphetamine users in the hope that targeted research can inform treatment services about how to better orient themselves towards this client group.

Treatment utilization amongst methamphetamine users interviewed in this study was low despite respondents claiming to be aware of treatment options. For example, while 96% of respondents were aware that counseling is available for methamphetamine dependence, only 22% of the sample had accessed counseling, and similarly only 15% of respondents had accessed inpatient withdrawal despite awareness levels at 78%. This indicates that awareness of treatment options is not the main reason for low rates of treatment engagement among methamphetamine users.

The three most common reasons identified for not having sought treatment all indicated that methamphetamine users did not think their use was serious enough or problematic enough to warrant formal treatment, despite these users having met DSM-IV criteria for methamphetamine dependence at some stage during their use. Furthermore, a common barrier to treatment was that methamphetamine users simply wanted to keep using. These findings suggest that methamphetamine users might not be accessing treatment because: a) they do not feel that they are dependent (i.e., DSM-IV criteria doesn't correlate with their own perceptions of dependence), b) they don't feel that regular use of methamphetamine warrants formal treatment, c) they discount their dependence, or d) they recognize their dependence but are not ready to do anything about it due to the benefits they receive from their drug use. A focus on harm reduction (emphasizing things such as improving physical health or psychosocial problems), might be more useful for methamphetamine users who are using heavily but not interested in formal treatment.

High levels of polydrug use were evident in the sample, and although methamphetamine was cited as the most common drug of choice among participants, there were also high rates of cannabis, heroin and alcohol among the sample. All of these drugs have sedative and/or depressant properties, which have the opposite effect to stimulant drugs such as methamphetamine. This may indicate that methamphetamine users either combine such drugs to compliment or interact with the effects of methamphetamine or use these drugs when they are not using methamphetamine to help with symptoms of withdrawal. Nevertheless this indicates that treatment for methamphetamine should incorporate a focus on other drug use.

For those methamphetamine users who sought treatment, the main reasons included being sick of the lifestyle, pressure from family and friends and seeking abstinence. These methamphetamine users were most likely to access counseling and inpatient withdrawal treatment, followed by Narcotics Anonymous. Barriers to effective service delivery for illicit drugs users more generally include social stigma, perceptions about treatment effectiveness, accessibility and long waiting periods [8-12]. These were all barriers identified by participants in this study, particularly issues around stigma and treatment effectiveness which were cited as two of the main barriers. Barriers identified by Wright, Klee and Reid from over 10 years ago [13] that include methamphetamine users not identifying themselves as 'hard' or dependent drug users and being unwilling to mix with heroin users, were not as evident in this study. However, some methamphetamine users did emphasize the 'opiate-centric' nature of treatment and indicated a desire for specialist treatment, or at the very least more specialized training for staff who do not have sufficient knowledge around the specific issues associated with methamphetamine.

Barriers to methamphetamine withdrawal treatment among service providers identified by Pennay and Lee [15] were different from those of users in this study. For example, mental health co-morbidity was not identified as a problem, nor was the 'binge' nature of use. However, methamphetamine users did note that waiting periods (resulting in drop out from treatment) and lack of a specific pharmacotherapy were barriers to treatment. Other barriers included the perceived negative attitudes of staff, which appears to be confirmed by the Pennay and Lee [15] study in which treatment staff voiced general pessimism towards these clients.

Respondents preferred to undergo withdrawal from methamphetamine on their own rather than seeking professional help, perhaps due to the perceived negative attitudes of staff and inappropriateness of services. Self-detoxification was the most frequent method of withdrawal from methamphetamine cited by participants, who frequently self-medicated to manage symptoms. This is consistent with previous research identifying self-detoxification as common among stimulant users [21]. Self-help materials to support and assist methamphetamine users through withdrawal may be useful in reducing the risks associated with self-withdrawal (including the concurrent use of other drugs), and could indicate the point at which users should consider seeking professional assistance if withdrawal becomes too difficult.

Other barriers noted included problems with availability, such as waiting lists and limited treatment spaces. One recent advancement towards improving accessibility to methamphetamine treatment is the development of a range of online services for drug use and mental health problems [22]. Such services may offer a 'soft entry' to formal treatment as well as important harm reduction and self-help information for methamphetamine users.

Other barriers included the belief that methamphetamine treatment is inappropriate, ineffective and incompatible with their personal goals. Methamphetamine users are asking for more efficient access to services that are better oriented to their needs. Such needs include counseling specific to methamphetamine use, a pharmacotherapy to ease withdrawal symptoms and specialist centers that are more adequately equipped to treat methamphetamine users. A treatment setting specific to methamphetamine users was identified in this study as one way to accommodate the very different nature of methamphetamine dependence and withdrawal compared to other drugs such as opiates.

In an attempt to overcome the well-established barriers to methamphetamine treatment, four stimulant specific clinics have been developed in Australia. In 2006, two specialist methamphetamine clinics, known as 'The Stimulant Treatment Program' (STP), were established in Sydney and Newcastle. An evaluation of the first six months of the STP clinics found that almost half of the 87 clients had not previously sought formal treatment for their methamphetamine use. Follow-up interviews with clients showed that clients had reduced their drug use considerably over the course of treatment, as well as severity of dependence, distress, mental health problems and crime, together with improvements in social functioning. This preliminary evaluation showed that the STP was feasible and was able to attract methamphetamine users into treatment, particularly those who weren't attracted into mainstream treatments [23].

In 2007, two specialist methamphetamine clinics ('Access Point') were established in the Melbourne suburbs of Fitzroy and St Kilda. The Access Point model was designed to address many perceived barriers to methamphetamine treatment, including providing timely service (i.e., no waiting periods), providing a waiting area separated from other clients (addressing barriers around methamphetamine users not wanting to mix with heroin users) and providing specialised and tailored methamphetamine treatment which included access to a counselor, psychiatrist and medical practitioner. An evaluation of the clinics over the first twelve months of operation showed that Access Point clients differed demographically from clients receiving standard AOD treatment, including being older, more likely to be employed and more likely to be smoking methamphetamine than injecting. Access Point showed better outcomes in term of treatment retention than standard treatment. In addition, Access Point clients showed good reductions in methamphetamine use and mental health problems [24].

Although establishing an entirely separate clinic is beyond the means of many drug treatment services, services can undertake small changes that could have a large impact on perceptions, such as allocating some time each day (for example, 3-5 pm) in which only methamphetamine users are seen or allocating specific staff or rooms to methamphetamine treatment that contain methamphetamine specific information and resources. The former would also reduce waiting times, another barrier to methamphetamine users entering treatment, as there would be a time in which these clients could be booked in within a day or two.

This study has identified some barriers to methamphetamine treatment. However, we have identified a number of ways in which treatment can be improved for methamphetamine users, including access to treatment through online programs, allocating a certain amount of time or treatment space to methamphetamine users and improving capacity of clinicians to be able to better respond to methamphetamine users. Finally, more research needs to be undertaken to identify an appropriate pharmacotherapy to either assist in the reduction of methamphetamine withdrawal symptoms or to act as a substitution treatment.

## Competing interests

The authors declare that they have no competing interests.

## Authors' contributions

PK, AH and AP were involved in collection of data, analysis of data and preparation of the manuscript. NK designed and coordinated the project and was involved in the preparation of the manuscript. All authors read and approved the final manuscript.
